# Development of a clinical feeding assessment scale for very young infants in South Africa

**DOI:** 10.4102/sajcd.v63i1.148

**Published:** 2016-10-26

**Authors:** Mari Viviers, Alta Kritzinger, Bart Vinck

**Affiliations:** 1 Department of Speech-Language Pathology and Audiology, University of Pretoria, South Africa

## Abstract

**Background:**

There is a need for validated neonatal feeding assessment instruments in South Africa. A locally developed instrument may contribute to standardised evaluation procedures of high-risk neonates and address needs in resource constrained developing settings.

**Objective:**

The aim of the study was to develop and validate the content of a clinical feeding assessment scale to diagnose oropharyngeal dysphagia (OPD) in neonates.

**Method:**

The Neonatal Feeding Assessment Scale (NFAS) was developed using the Delphi method. Five international and South African speech-language therapists (SLTs) formed the expert panel, participating in two rounds of electronic questionnaires to develop and validate the content of the NFAS.

**Results:**

All participants agreed on the need for the development of a valid clinical feeding assessment instrument to use with the neonatal population. The initial NFAS consisted of 240 items across 8 sections, and after the Delphi process was implemented, the final format was reduced to 211 items across 6 sections. The final format of the NFAS is scored using a binary scoring system guiding the clinician to diagnose the presence or absence of OPD. All members agreed on the format, the scoring system and the feeding constructs addressed in the revised final format of the NFAS.

**Conclusion:**

The Delphi method and the diverse clinical and research experience of participants could be integrated to develop the NFAS which may be used in clinical practice in South Africa or similar developing contexts. Because of demographically different work settings marked by developed versus developing contexts, participants did not have the same expectations of a clinical dysphagia assessment. The international participants contributed to evidence-based content development. Local participants considered the contextual challenges of South African SLTs entering the field with basic competencies in neonatal dysphagia management, thereby justifying a comprehensive clinical instrument. The NFAS is aimed at clinicians working in Neonatal Intensive Care Units where they manage large caseloads of high-risk neonates. Further validation of the NFAS is recommended to determine its criterion validity in comparison with a widely accepted standard such as the modified barium swallow study.

## Introduction

Clinical assessment is an important part of evidence-based management of neonatal dysphagia (Thoyre, Park, Pados & Hubbard, 2013). The purpose of clinical assessment is to establish the possible nature of the feeding problem, to explore the parent’s perception of the problem and the neonate’s readiness for oral feeding, to make a differential diagnosis and to determine the need for multi-disciplinary management (Arvedson, 2008; Rommel, 2006; Thoyre *et al*., 2013). The two main components of such an assessment include a parent interview and medical chart review – to obtain the feeding, medical and developmental history – as well as the clinical feeding assessment (Arvedson, 2008; Lau & Smith, 2011). With the development of a novel clinical assessment instrument, the researchers acknowledge the importance of comprehensive clinical assessment, but concurs with studies (Arvedson, 2008; De Matteo, Matovich & Hjartarson, 2005; Rommel, 2006) that clinical assessment is not designed to replace objective instrumental assessment such as the modified barium swallow study (MBSS). A clinical instrument should support an accurate diagnosis and description of the feeding profile related to oropharyngeal dysphagia (OPD) in high-risk neonates. The use of validated instruments should be encouraged in clinical practice because it provides a common language among clinicians, facilitates the production of diagnostic data and promotes the evaluation of techniques and approaches used during clinical assessment (Brandao, Dos Santos & Lanzilotti, 2013).

There is a high prevalence of low birth weight (LBW) and prematurity in South Africa (WHO, 2012) contributing to neonatal OPD. In the USA, the prevalence of feeding disorders in premature neonates is estimated between 10.5% and 24.5% (Jadcherla, 2016). Currently, no prevalence figures on feeding disorders associated with prematurity are available in South Africa. The high prevalence of feeding disorders among the neonatal population supports the need for appropriate early clinical assessment and management of OPD, providing an impetus for the development of a valid clinical instrument to contribute to differential diagnosis. In the South African public healthcare sector, there are resource constraints such as limited or no speech-language therapists (SLTs) to provide feeding services in some Neonatal Intensive Care Units (NICUs) (Strasheim, Kritzinger & Louw, 2011). SLTs working in hospitals are also required to manage large caseloads apart from neonatal dysphagia and then do not have the opportunity to specialise in the field. In addition, inexperienced community service therapists are frequently the only service providers in some settings (Singh *et al*., 2015).

Existing dysphagia assessment instruments may not meet the needs in South Africa. Philbin and Ross (2011) developed the ‘support of oral feeding for fragile infants’ (SOFFI) which includes a systematic approach to assessment of bottle feeding and clinical decision-making for intervention. The Department of Health in South Africa promotes the World Health Organization guidelines (WHO, 2010) for infant feeding which recommend exclusive breastfeeding for the first 6 months of life (National Department of Health, 2015). The bottle-feeding approach of the SOFFI therefore has limited application in the healthcare sector in South Africa. Some reliable clinical instruments that are supported by high-level evidence do exist, but do not focus holistically on neonatal feeding. The Neonatal Oral Motor Assessment Schema [NOMAS] (Palmer, Crawley & Blanco, 1992) and the Schedule for Oral Motor Assessment [SOMA] (Reilly, Skuse & Wolke, 2000) both focus on oral motor skills only (Pressman, 2010; Rogers & Arvedson, 2005). These two scales do not address a feeding assessment from a bio-psychosocial perspective to diagnose OPD. Such a perspective acknowledges the impact of NICU environmental stressors on state regulation, internal physiological disruptions on the neonate’s subsystems and the resulting effects on the feeding process, as well as mother–infant interaction during feeding. A clinical assessment instrument should assist the SLT to assess all neonatal systems that contribute to and interact with the feeding process. The instrument should consider the sequential development of the sensory systems emerging throughout gestation in a developmentally supportive approach (Browne & Ross, 2011; Thoyre, 2007). Such an instrument should also be comprehensive to facilitate the description of symptoms related to sensory and motor-based feeding difficulties (Lau & Smith, 2011) that may result in OPD from 32 weeks gestational age. Neonatal OPD is any interference with the acts of feeding and/or swallowing that interrupts the oral or pharyngeal stage of swallowing compromising the development of typical feeding and swallowing skills and the neonate’s nutritional and respiratory status (Arvedson, 2008; Browne & Ross, 2011; Rogers & Arvedson, 2005). The condition is typically only diagnosed from 32 weeks gestational age when nutritive sucking (NS) should emerge (Rogers & Arvedson, 2005; Thoyre, 2007). To facilitate the assessment process, an instrument should provide prompts for observation of a variety of signs and symptoms related to neonatal OPD.

The purpose of neonatal feeding assessment is to accurately diagnose OPD to prevent the negative sequalae of OPD. Such negative effects may include inadequate weight gain, dehydration, and limited oral sensory experience, which may continue to impact on infancy and early childhood. Obtaining expert opinions on such a new instrument would be invaluable for the development and validation process. This article will report on experts’ opinion on the development of the content and face validity of a clinical feeding assessment instrument.

## Method

### Aims

The aim was to develop and validate the content of a novel clinical feeding assessment scale to diagnose OPD in neonates. The objectives to support the aim were (1) to determine if the panel of experts agreed about the need for a validated clinical feeding assessment scale, (2) to select appropriate items for inclusion in the Neonatal Feeding Assessment Scale (NFAS) and lastly (3) to establish face and content validity of the NFAS based on expert input.

### Design

The Delphi method (Hassan, Keeney & McKenna, 2000) was used to gather quantitative and qualitative data from an expert panel during two rounds of consecutive questionnaires. Qualitative data were obtained from open questions, and quantitative data from closed questions. The Delphi method was used to guide improvement of content and face validity of the new instrument. This method allowed the researchers to investigate whether the NFAS represented all facets of neonatal feeding skills. The primary strength of the Delphi method is the objective exploration of issues that require judgement, such as the content and measurement methods when developing a clinical assessment instrument. Because the Delphi method is considered one of the most commonly used research procedures to establish content validity of an assessment instrument by an expert panel (Hassan *et al*., 2000), this design was considered suitable for the purpose of this study.

## Participants

Five expert panel members were included in the study. Informed consent was obtained from all participants. Participant selection criteria included a Masters’ degree qualification in speech-language pathology from an accredited tertiary institution to guarantee a high level of expertise and at least 5 years clinical experience in the field of paediatric dysphagia. Participants could reside in South Africa or internationally. In Table 1, a summary of participant characteristics is provided.

**TABLE 1:** Participant description (*n* = 5).

Characteristics	Number of participants
**Gender**	
Female	5
Male	0
**Years of working experience**	
5–10 years	1
10–20 years	1
>20 years	3
**Working context**	
Public health care	1
Private health care	1
Academic and public healthcare	2
Other: Non-governmental organisation providing clinical services	1
**Citizen country**	
South Africa	3
USA	2
**Qualification**	
Master’s degree	2
Doctoral degree	3

All participants had postgraduate qualifications in the field of speech-language pathology. Both international experts had doctoral degrees in paediatric dysphagia which demonstrated their advanced knowledge. In addition, the international experts had more than 20 years of clinical experience working in paediatric dysphagia. This highlighted the long history of paediatric dysphagia intervention in the USA as well as the experts’ significant clinical experience. Only one of the South African participants had more than 20 years’ clinical experience.

## Materials

The NFAS will not be described in detail in this section because the purpose of the study was to develop and validate the content of the instrument. The NFAS was based on other clinical assessment instruments, studies on neonatal feeding development, relevant literature on prematurity, LBW and paediatric HIV and/or AIDS in the South African context and recent studies on neonatal dysphagia. Additionally, the first author’s clinical experience of service delivery in the private and public healthcare sectors in the NICU provided insight into local needs and knowledge of specific local constraints.

Two self-composed electronic questionnaires were used to obtain feedback from the expert panel on the content of the NFAS. Round one required a comprehensive overview of the NFAS and round two required targeted responses in closed question format about the revised content, structure and format of the NFAS. The two questionnaires contained questions on the relevance of separate sections and items relating to the different neonatal systems involved in feeding in the NFAS. Both questionnaires gave the participants the opportunity to offer recommendations on the addition or removal of sections and items, to comment on different scoring methods, and to judge the comprehensiveness of the scale and its relevance to clinical use in hospitals. Open-ended and some close-ended questions were also included addressing face validity, user friendliness, and the format of the instrument and technical editing (Dawson & Trapp, 2004). For close-ended questions, reasons for answers had to be given. The questionnaires facilitated a deductive reasoning sequence to compile an authentic profile of neonatal feeding skill assessment. The first questionnaire focused on the content domains of skills related to neonatal feeding and swallowing (Als *et al*., 1994; Arvedson, 2008; Arvedson & Brodsky, 2002; Bahr, 2001; Brazelton, 1973; Browne & Ross, 2011; Clark, 2009; Da Costa & Van der Schans, 2008; Darrow & Harley, 1998; Dieckmann, Brownstein & Gausche-Hill, 2006; Gewolb & Vice, 2006; Hall, 2001, 2011; Henning, 2002; Hodgman, Hoppenbrouwers & Cabal, 1993; Jadcherla, 2016; Karl, 2004; Nugent, 2007; Prechtl & Beintema, 1964; Qureshi, Vice, Taciak, Bosma & Gewolb, 2002; Rudolph & Link, 2002; Swigert, 2010; Tsai, Chen & Lin, 2010; Van Haastert, De Vries, Helders & Jongmans, 2006; Wolff, 1959; Wolf & Glass, 1992) – see Table 2. A draft version of the NFAS accompanied the first questionnaire.

**TABLE 2:** Content and rationale for expert panel questionnaire 1.

Questions	Rationale for including item in questionnaire
Question 1.1–1.9: Do you consider the following section included in the NFAS to be comprehensive enough to obtain adequate information during a clinical assessment of a high-risk neonate’s feeding skills?	To determine if the main components related to the construct of neonatal feeding are included in the different sections of the draft of the NFAS.
Question 2.1–2.9: Do you consider the following item/s included in the NFAS to be comprehensive enough to obtain adequate information during a clinical assessment of a high-risk neonate’s feeding skills?	To determine if the items in each proposed section addressed the main components related to the construct of neonatal feeding.
Question 2.1–2.2: If you select ‘no’ for any particular item/section, motivate your choice and indicate items/sections to be added or omitted.	Participants could comment and reason about the relevance of components, sections and items that investigates neonatal feeding skills.
Question 3: Comment further on the sections and items in the NFAS if all your opinions/suggestions could not be expressed in the previous questions.	Additional information could be offered that may not have been included by the preceding closed questions.
Question 4: Is the development of a validated clinical assessment instrument a relevant area of study?	To obtain the participants’ opinion on the need and relevance for developing a neonatal dysphagia assessment instrument.
Question 5: Is there a need for the development of a validated clinical assessment instrument to use in clinical practice with neonatal dysphagia in the international arena?	To determine the international need for such a tool.
Question 6.1–6.5: Please provide your opinion and recommendations regarding the following components of the NFAS: 6.1 Scoring method 6.2 Face validity 6.3 Professional appearance 6.4 User friendliness 6.5 Language and technical editing	6.1 To receive feedback on the proposed scoring method of the NFAS. 6.2–6.5 All aspects of face validity were included (Meline, 2010).

NFAS, Neonatal Feeding Assessment Scale.

The second questionnaire was developed based on the responses and feedback obtained in the first questionnaire. The NFAS was adapted according to the experts’ feedback. The revised NFAS was then sent to the expert panel along with the summary of changes recommended in the first round. The second questionnaire was used to further refine the content and face validity of the instrument (Table 3).

**TABLE 3:** Content and rationale for expert panel questionnaire 2.

Question	Rationale for inclusion
1. The revised instrument is user friendly	To allow the participants to judge relevant components (sections and items) of the revised NFAS that should be considered in the final format of the instrument.
2. The format and technical editing of the revised instrument is acceptable	
3. The face validity of the revised instrument is acceptable	
4. The proposed scoring system of the revised instrument is acceptable	
5. The revised feeding constructs for the identified target population is acceptable	
6. The content validity of the revised instrument is acceptable	
7. Provide additional comments on the revised instrument	To provide an opportunity to the participants to give additional comments if they were of the opinion that a component was not sufficiently addressed with the questions posed in both questionnaires.

NFAS, Neonatal Feeding Assessment Scale.

## Procedures

**FIGURE 1:**
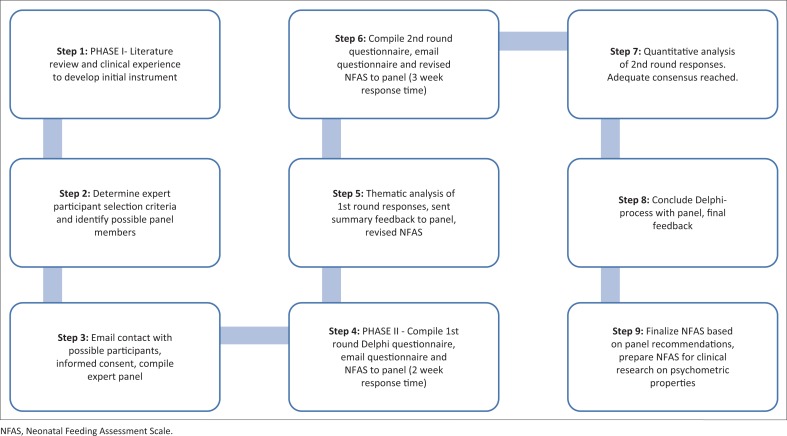
Flowchart of study procedures.

Clearance was obtained from the research ethics committee at the university where the study was conducted. The process of validation of a new assessment instrument commences with the initial development phase providing a sound theoretical foundation to link to clinical practice (St Pierre *et al*., 2010). The initial phase of instrument development consisted of the review of available published scales, checklists and literature, and the researchers’ own clinical experience. The second phase employed the Delphi method to request expert judgement on the new clinical instrument. The panel members’ identity was blinded to one another to enhance open participation in the instrument development process. The procedures followed in the study are depicted in Figure 1.

The preliminary and revised instrument was sent to the expert panel to facilitate two rounds of questioning via email. The panel was blind to one another’s responses. The aim of the first round was to allow the expert panel to judge the validity of the content domains in the instrument. Summarised feedback, to the panel, after round one served as the introduction of round two. The aim of the second round was to reach consensus on the recommendations of the first round, as well as on the content and the scoring system of the instrument. After the second round responses were received from the participants, the Delphi process was concluded, as majority agreement and no new additional content was suggested, indicating that adequate consensus among panel members had been reached. The Delphi method allowed rich data to be gathered because open and closed questions could be used to probe the participants’ views on the NFAS. Round one rendered descriptive data which was analysed according to emerging themes linked to the various content sections of the draft instrument.

## Data analysis

According to Hassan *et al*. (2000), the Delphi method is not intended to produce statistically significant results, but rather a synthesis of an expert group’s opinion. Suggested changes according to the themes that emerged from the data will be discussed. Sections of the data of round one and all of the data from round two were analysed quantitatively using frequency counts.

## Results

Results will be presented according to the three objectives of the study.

### Objective A

#### Determining agreement on the need for a validated feeding assessment instrument

Three themes were identified linked to the content sections of the first questionnaire. The first content theme was the *Need for a valid assessment tool.* The second theme was *Content of the NFAS* and the last was *Scoring criteria.* Only the first theme’s results are discussed with this objective. Questions 4 and 5 in the first questionnaire investigated the rationale for the development of the NFAS. All participants (*n* = 5; 100%) agreed that the development of a valid clinical assessment tool was a relevant area of study and confirmed the need for such a tool. Some participants also provided further comments to reflect their agreement.

It was stated that:

‘…there is definitely a need for a well-researched assessment tool for use with infants…’ [Participant 4, Female, SLT]

However:

‘internationally still a huge lack of normative data regarding sucking and swallowing along with more global developmental aspects of feeding in young infants….difficulty lies in subjectivity of observation of skills that are not measurable…’ [Participant 2, Female, SLT]

One of the South African panel members commented that:

‘…in South African public healthcare an instrument would help with prioritisation of a large case load on assessment outcomes that are valid…and prevent over referral to video swallows….’ [Participant 5, Female, SLT]

In addition, one of the participants stated that a validated feeding assessment instrument might support clinicians in case management. The qualitative comments further supported the rationale for research to develop a validated feeding assessment instrument for use with the neonatal population.

### Objective B

#### Content and item selection of the Neonatal Feeding Assessment Scale

The content and item selection of the preliminary NFAS was based on theoretical constructs related to neonatal feeding and the clinical assessment of feeding difficulty in early infancy. The instrument relies on physiological observations of the neonate during feeding and elicitation of oral responses. Neonatal states were included so that the influence on feeding and state disruption as a result of feeding difficulty may be observed. The structure of the initial draft of the NFAS included three different age categories – from 32 weeks gestational age to 4 months corrected age post term. These different age categories allowed for the inclusion of developmentally appropriate items. In Table 4, the content of the NFAS and the rationale for content selection are summarised.

**TABLE 4:** Preliminary Neonatal Feeding Assessment Scale content and rationale for item selection.

Sections	Rationale	References
A: Physiological subsystem functioning	Because respiratory problems are one of the most common causes of paediatric dysphagia, assessment of respiratory patterns during feeding was included. Respiratory rate and heart rate may further reveal signs of dysphagia and possible chronic aspiration. Airway stability is a prerequisite for successful oral feeding.	Als *et al*., 1994; Arvedson, 2008; Dieckmann, Brownstein & Gausche-Hill, 2006; Hall, 2001; Hodgman, Hoppenbrouwers & Cabal, 1993
B: State of alertness during feeding	As neonate’s state typically varies during feeding, behaviour should be assessed to determine the optimal stage of alertness to proceed with oral feeding. The neonate should be in an optimal state of alertness for successful oral feeding. The different stages of alertness and subsequent impact on feeding ability were informed by the synactive theory of development.	Als, 1982; Arvedson & Brodsky, 2002; Brazelton, 1973; Nugent, 2007; Prechtl & Beintema, 1964; Wolff, 1959
C: Stress cues during feeding	A neonate’s ability to respond to incoming sensory information plays a role in feeding readiness. Interaction between state regulation, the motor system and the autonomic nervous system should be observed to determine stress during feeding and to enable the clinician or parent to make adaptations.	Als, 1982; Brazelton, 1973; Hall, 2001; Karl, 2004; Tsai, Chen & Lin, 2010
D: General movement and muscle tone screening	Adequate postural control is a prerequisite for safe and efficient feeding. Inadequate muscle tone, postural control or movement may impact negatively on oral feeding. If difficulties are observed, referral to an occupational therapist and/or a physiotherapist can be made.	Arvedson & Brodsky, 2002; Clark, 2009; Hall, 2001; Van Haastert, De Vries, Helders & Jongmans, 2006
E: Oral peripheral evaluation	Successful swallowing requires the coordination of 31 muscles and five cranial nerves. Neonatal anatomy, physiology, primitive oral reflexes and underlying cranial nerve function should be assessed.	Arvedson & Brodsky, 2002; Bahr, 2001; Hall, 2001; Henning, 2002; Swigert, 2010; Wolf & Glass, 1992
F: Clinical feeding and swallowing evaluation	The purpose of clinical assessment is to observe the oral preparatory and/or oral stage of swallowing and make certain inferences about the pharyngeal stage, provide baseline feeding and swallowing data for further management and to determine progress.	Arvedson, 2008; Arvedson & Brodsky, 2002; Da Costa & Van der Schans, 2008; Darrow & Harley, 1998; Gewolb & Vice, 2006; Hall, 2011; Jadcherla, 2016; Qureshi, Vice, Taciak, Bosma & Gewolb, 2002; Rudolph & Link, 2002; Swigert, 2010
G: Parent–neonatal interaction during feeding	Success with infant feeding depends on the parent/caregiver’s ability to monitor the neonate’s stress cues and to make environmental adaptations in order to facilitate success. At-risk neonates’ experience an increased potential for developing relational interaction difficulties. It is important to note that parent–infant interaction during feeding establishes a foundation for social communication interaction and the inherent reciprocity of the communication dyad.	Arvedson & Brodsky, 2002; Browne & Ross, 2011; Hall, 2001
H: Use of compensatory strategies	As part of initial assessment the clinician should be able to recommend compensatory strategies to support successful feeding in the neonate. Strategies to consider may include modifying the positioning of the neonate during breast/bottle feeding, type of bottle/nipple used or external pacing during breast/bottle feeding. These strategies may empower the mother to feel in control of the feeding process and may build her confidence in meeting her infant’s nutritional needs.	Arvedson & Brodsky, 2002; Hall, 2001; Swigert, 2010

All five participants contributed to both rounds of the Delphi process resulting in a 100% response rate. The results of rounds one and two are presented separately. The thematic analysis of the first theme of round one was discussed, and examples of panel member responses to complement the data were provided in the first section of the results; however, in this section results related to the second and third themes are presented. The closed question responses of rounds one and two are combined and will be presented in table format.

### Results of round one

The second theme addressed the *Content of the NFAS*, it was stated:

‘…it is a very comprehensive tool covering all necessary areas…’. [Participant 1, Female, SLT]

A similar comment was made by Participant 2. However, it was stated that:

‘….section G [parent-neonate interaction] and H [use of compensatory strategies] are not that relevant to first-time assessment…I view it as part of treatment already…consider removing it from the current instrument’. [Participant 5, Female, SLT]

Three of the participants indicated that these two subsections were too subjective and not directly relevant to initial assessment and diagnosis of OPD. These subsections were then omitted from the final instrument. Four participants also suggested revision of some of the items related to feeding and swallowing ability in the content domains in sections C, E and F. Based on some participant’s feedback (*n* = 3), there was support for the notion of a comprehensive clinical assessment in the neonatal stage, despite indicating that the instrument was too lengthy.

The recommended *scoring system of the NFAS* (theme three) included allocation of marks if a skill and/or behaviour was present or absent. The clinician would then calculate a score for each section and a final score for feeding difficulties to conclude the assessment. The higher the score, the more likely a neonate could be diagnosed with OPD. Theme three dealt with the *Scoring criteria.* Statements were made, such as:

‘…consider simplifying the scoring system for ease of use…might be confusing in current format’ and ‘[y]ou need to score a concept to compare it to a gold standard to be able to validate it’ [Participant 1, Female, SLT]

Another comment was:

‘…the scoring system will be easier if binary scoring in a checklist format is used in the final version of the instrument….with a good explanation of administration guidelines…’ [Participant 4, Female, SLT]

One of the South African participants stated:

‘the scoring system is a bit confusing in this format…instructions on how to assess the neonate should be expanded…since some speech therapists might lack experience….and need help…’ [Participant 5, Female, SLT]

Three participants suggested clearer administration guidelines and using a different approach to score the data. Results of round one led to the refinement of the initial scoring system. Binary choices were included for each item in all sections, with clear administration and scoring guidelines in the revised instrument. The scoring method was refined with assistance from a biostatistician to include a binary (yes/no) outcome for each section and a total score that will enable comparison with a widely accepted gold standard for swallowing assessment, in this case the MBSS.

In summary, all participants agreed on the need for more research to develop a validated assessment instrument. Three of the five participants agreed on the comprehensive nature of the proposed content for the draft NFAS. Lastly, all the participants recommended refinement of the scoring system.

However, differences in opinion encountered in the feedback from participants in round one were analysed further to highlight how the South African panel members’ responses differed from the international participants’ contributions. These differences may be as a result of the disparity of resources between the developing and developed context of the participants, and challenges experienced in the local context that international participants may not be aware of. A difference in opinion was clearly evident between the two groups of panel members about the length of the instrument and item inclusion of which both components related to the comprehensive nature of the NFAS.

### Results of round two

Upon conclusion of round one, the NFAS was revised according to recommended changes where the majority opinion (Dawson & Trapp, 2004) motivated the changes. To initiate round two, a summary of the first round’s recommendations and the revised instrument were sent to the participants.

### Objective C

#### Face and content validity of the final version of the NFAS

The second questionnaire provided quantitative data that could be compared with some of the close-ended questions in round one. Round two offered an opportunity for additional comments by the panel members if they felt that the previous round did not address all their concerns. The comparative results of the two rounds are depicted in Table 5.

**TABLE 5:** Quantification of degree of agreement among participants.

Question topic	Round one		Round two	
	Agree† (%)	Disagree† (%)	Agree† (%)	Disagree† (%)
The instrument/revised instrument is user friendly	60	40	80	20
The format and technical editing of the instrument/revised instrument is acceptable	60	40	100	0
The face validity of the instrument/revised instrument is acceptable	60	40	80	20
The proposed scoring system of the instrument/revised instrument is acceptable	0	100	100	0
All the subsections and items in the draft should be included in the final instrument	60	40	n/a	n/a
The revised feeding constructs for the identified target population is acceptable	n/a	n/a	100	0
The content validity of the revised instrument is acceptable	n/a	n/a	80	20

† *n* = 5.

According to Table 5, the majority of panel members’ (*n* = 4) opinions regarding some of the concepts probed in round one and again in round two (closed questions) reflected increased agreement on the probed components of the final version of the NFAS. One participant did not agree on the user friendliness, content and face validity in round one. To ensure scientific rigor, the Delphi process holds researchers accountable by providing a true account of the participation responses. As participants responded via email, data could be saved and verified. No qualitative comments were received in round two. According to Table 4, there were a number of disagreements in round one that was resolved in round two, which indicated high agreement among the panel. All members agreed on the format, the scoring system and the feeding constructs addressed in the revised final format of the NFAS.

The final content and checklist format of the NFAS, which resulted from the Delphi process, consisted of six sections with different items. The NFAS is summarised in Table 6.

**TABLE 6:** Overview of the final Neonatal Feeding Assessment Scale.

Sections	Subsections	Subsections removed from draft NFAS	Initial number of items	Revisions of the NFAS
A: Physiological functioning Subsections:	Heart rate Respiratory function (According to three age categories)	Colour of neonate’s skin	38 items	29 items (arranged according to gestational or corrected age ranges in both subsections). Nine items related to normal skin colour and skin discolouration were removed.
B: State of alertness during feeding	-	None	7 items	No changes
C: Stress cues during feeding Subsections:	State-related stress cues Motor-related stress cues Autonomic-related stress cues (graded as mild, moderate or severe)	None	43 items	Reduced to 35 items, removing eight items related to various stress cues: State-related stress cues: removed four items such as ‘discharge smiling’, ‘eye-floating’, ‘gaze aversion’ and ‘glassy-eyed’. Motor-related stress cues: removed one item namely, ‘facial grimacing’. Autonomic-related stress cues: two moderate cues (bowel movement & multiple swallows) were removed together with one severe cue, namely ‘reflux’.
D: General movement and muscle tone screening Subsections:	At rest During feeding (According to three age categories)	None	17 items	Reduced to 12 items (arranged according to gestational or corrected age ranges in both subsections). Four items related to a conclusion about general muscle tone were removed and one item related to ‘independent head support’ that was not developmentally appropriate for the age ranges. Remaining items were reorganised related to observations at rest and during feeding in the various age categories.
E: Oral peripheral examination Subsections:	Oral reactions Oral structure and function Observation of cranial nerve function to indicate symptoms of possible dysfunction	Physical symptoms of illness	45 items	Increased to 72 items. A subsection’s name was changed to ‘Observation of cranial nerve function to indicate symptoms of possible dysfunction’ was based on recommendations by the participants. Various symptoms in the subsection of cranial nerve function were separated for scoring generating an increase of 12 items. Two items related to symptoms of physical illness were removed. In the subsection of oral structure and function items in subcategories related to the lips, cheeks, palate, tongue and jaw at rest and during feeding were refined generating an increase of 18 items in this subsection.
F: Clinical feeding and swallowing evaluation Subsections:	NNS: according to two age categories NS: according to two age categories Behavioural response to feeding and non-nutritive sucking stimulation Symptoms of OPD (NNS and NS are evaluated according to the different age categories)	Saliva management Feeding methods Tactile response to NNS and NS Positioning	90 items	Reduced to 56 items (items in the NNS and NS subsections are arranged according to gestational or corrected age ranges). Rephrasing of some items. Three items were removed in the saliva management subsection. The subsection on NNS was separated into two age categories and further refinement in the two categories generated six additional items. The NS subsection was also separated into the same two age categories increasing items from 8 to 32. An integrated subsection was created from two previous subsections, namely ‘Avoidance behaviour during NS’ and ‘Infant’s behavioural response to feeding method’. The new subsection was, ‘Behavioural response to feeding method and NNS stimulation’. This integration reduced 26 items to five remaining items. The subsection of ‘Positioning’ was incorporated in subsection D. The subsection on ‘Pharyngeal dysphagia’ was changed to include ‘Symptoms of oropharyngeal dysphagia’ including two subcategories representing 14 items.

NFAS, Neonatal Feeding Assessment Scale; NNS, non-nutritive sucking; NS, nutritive sucking; OPD, oropharyngeal dysphagia.

All the changes were made based on majority recommendations of the expert panel. According to Table 6, one subsection in Section A contained nine items relating to the discolouration of the neonate’s skin indicating lack of oxygen in the orofacial area. The majority of participants considered these items too subjective for accurate scoring, and therefore, it was removed. Section B remained unchanged because participants suggested no changes. In Section C, eight items relating to various stress cues were removed because of possible ambiguity, repetitiveness or vagueness indicated by three participants. Section D was reduced from 17 to 12 items to screen muscle tone and movement in a more concise manner because five of the items were considered redundant by four participants. In the last two sections (E and F), items suggested by all the participants were added to ensure comprehensive observations of oral structure as well as neonatal feeding and swallowing skills. However, the international panel members recommended that subsections (in Sections E and F) relating to physical symptoms of illness (e.g. oral thrush in neonates with HIV and/or AIDS), saliva management and feeding methods should rather be obtained from the neonate’s medical record or during the parent interview, and therefore, it was removed. In some of the subgroupings in Sections E and F, where feeding skills relate to developmental level, two age categories were linked to assessment items and criteria leading to a reorganisation of items. All the participants agreed on the use of these age categories. Age categories may enable serial assessment to build a feeding profile over time whilst the neonate is receiving hospital-based care.

The components of comprehensive clinical feeding assessment that emerged were the observation of physiological status, state of alertness, stress cues, postural control and tone related to feeding position, oral-motor structure and function, non-nutritive sucking (NNS) and NS, behavioural responses to feeding and symptoms of OPD (Dodrill, Cleghorn, Donovan & Davies, 2008; Lau & Smith, 2011; Thoyre *et al*., 2013). These components were all addressed in the revised NFAS. The length of the instrument relates to the local need and aim of a comprehensive assessment tool which should include signs and symptoms reflecting the presence of OPD in neonates.

## Discussion

### Need of the Neonatal Feeding Assessment Scale

The need of a clinical tool to assess OPD in high-risk neonates was established. In a review of oral feeding assessment instruments for infants younger than 6 months, the findings of Pados *et al*. (2016) support the identification of this need. They concluded that there is a need for the development and testing of feeding assessment tools for young infants to guide optimal clinical practice. It is also suggested that such assessment tools should allow use for breast and bottle feeding for consistent assessment across feeding methods. Meeting this need may facilitate more appropriate management of OPD in neonates, because intervention will be guided by reliable and comprehensive assessment findings with an accompanying diagnosis. Infants discharged with inadequate investigation into the feeding difficulties or unresolved feeding difficulties, LBW and prematurity are more at risk of developing failure-to-thrive than their term counterparts with appropriate weight for age (Browne & Ross, 2011). Valid and reliable assessment instruments will help clinicians to objectively evaluate feeding (Pados *et al*., 2016).

## Development, face- and content validity of the Neonatal Feeding Assessment Scale

The Delphi method was used to develop the final format of the NFAS and to establish face and content validity. This was achieved by convening an expert panel to assist with the further development of a novel clinical feeding assessment instrument. The interaction process was collaborative and yielded constructive comments supporting the validation of the NFAS. The Delphi process was helpful to consider appropriate feeding constructs for content selection, to develop a reliable scoring system and to enable transparency and replication of methodology. Differences in opinion between the local and international participants emerged and may likely be ascribed to the working context in developing versus developed countries, emphasising the challenges present in the South African context. The participants’ comments supported the rationale of the study regarding the development of a neonatal feeding assessment instrument supported by evidence, but also highlighted the subjective nature of observation of skills related to neonatal feeding. This calls for more research on objective measurement of skills related to feeding difficulties in neonates.

The South African participants did not see a need to shorten the NFAS significantly because they felt that it ensures holistic and comprehensive clinical assessment that might be lacking in inexperienced clinicians. In contrast, the international participants were of the opinion that the instrument was too lengthy for clinical use in the initial version. This may be due to the international experts being more experienced than some of the South African participants in clinical practice, because both of the international experts had more than 20 years’ experience working in the field of paediatric dysphagia. The participant responses assisted the researchers in refining the content and items of the NFAS.

South African participants considered comprehensiveness as important in clinical service delivery in resource constrained settings. Many inexperienced clinicians are conducting their community service year and require guidance. A comprehensive assessment instrument may prompt observations which may be missed when item descriptions are omitted. International participants focused on the subjectivity of some items which revealed that they were more experienced and therefore concerned with the levels of evidence to support the inclusion of sections and items, especially in a context where inexperienced SLTs may be using the NFAS. No difference in opinion regarding the scoring criteria and guidelines was noted. However, one international participant was the only expert who recommended consultation with a biostatistician, demonstrating knowledge of instrument development acquired during her research career.

Owing to demographically different work settings marked by developed versus developing contexts impacting on healthcare service delivery, participants did not have the same expectations of a clinical assessment. The local participants were aware of inexperienced SLTs entering the public health system in their community service year and having to diagnose OPD without MBSS equipment. The NFAS was designed to prompt inexperienced SLTs to include appropriate content domains during clinical assessment and supports a comprehensive approach to assessment of neonatal feeding problems such as OPD. Paediatric and adult dysphagia were formally included as a module in undergraduate Speech-Language Pathology curricula in 2004 in South Africa (see Faculty of Humanities Undergraduate Syllabi and Regulations, 2004, University of Pretoria as an example). There is thus only an 11-year history of formal professional training at universities in South Africa. Although dysphagia is now an established component of local Speech-Language Pathology curricula, much research is still required. Dysphagia is a relatively new, yet growing field in the profession in South Africa with active pursuit of research (Blackwell & Littlejohns, 2010; Pike, Pike, Kritzinger, Krüger & Viviers, 2016; Singh *et al*., 2015).

## Outcome of the Delphi process

The participants had the opportunity to critically evaluate the revised NFAS as indicated by their change in responses in round two, leading to majority consensus (see Table 3). One of the members who did not agree on the user friendliness of the draft instrument still indicated that the NFAS was too lengthy despite revision. This concern already emerged in round one and was addressed through implementing the recommended changes (see Table 6) and using a checklist format that improved effectiveness. The same participant indicated that the face and content validity were not completely adequate because many observations remained subjective in nature. The researchers attempted to include measurable items where possible to decrease subjectivity; however, this was not possible for all items. There remains a great need for further research on neonatal feeding skills and objective measurement technologies. The validity of content and items were supported by using current research on developmental skills and feeding abilities of neonates. The aforementioned concerns were addressed as far as possible in the final format of the NFAS. When interpreting results in a Delphi process, the majority opinion motivated the changes, but if a valid contribution is offered by a single participant or a minority, the researchers may choose to use it (Okoli & Pawlowski, 2004). In the revised NFAS, local needs were paramount and the South African participants preferred a comprehensive assessment instrument.

Similar to the NFAS, other researchers in health sciences also found the Delphi method useful in contributing to the successful development of clinically relevant assessment instruments (Crist, Dobbelsteyn, Brousseau & Napier-Phillips, 2004; Da Costa, Van den Engelhoek & Bos, 2008; Schulz *et al*., 2009; Yousuf, 2007). The NFAS is aimed at clinicians working in NICUs, where they manage large caseloads of very young high-risk populations. An increased prevalence of high-risk neonates exists in developing countries such as South Africa (WHO, 2012). Early identification of OPD whilst these neonates are still accessible in the hospital is important to allow opportunity to train mothers to manage feeding difficulties before discharge. In addition, OPD appears to be more prevalent than growth problems in preterm neonates and is likely to continue into early childhood, thereby indicating the need for early intervention to address feeding difficulties and minimise caregiver stress (Crist *et al*., 2004).

The NFAS aims to provide a developmentally supportive approach to assessment as proposed by Thoyre *et al*., (2013). The NFAS is minimally invasive because assessment is mainly through observation of a broad scope of skills before and during feeding to prevent overloading neonatal sensory systems with physical handling. Studies by Philbin and Ross (2011) as well as Browne and Ross (2011) indicated that unnecessary physical handling may disrupt state regulation during this sensitive stage of neurological development. Another characteristic of the NFAS includes the parent/caregiver in family-centred service delivery. Mothers contribute greatly to feeding assessment by providing information about their infant, and their experience and feelings surrounding the feeding challenges. A family-centred developmentally supportive approach relates to current evidence in the field of neonatal dysphagia (Lau & Smith, 2011; Thoyre *et al*., 2013).

## Conclusion

In South Africa, the field of paediatric dysphagia was formally introduced to curricula at universities in 2004, but was practiced many years prior to this introduction. Issues such as resource constraints, inadequate infrastructure, new graduates required to manage large caseloads in the public health system, few expert clinicians in practice and feeding difficulties related to HIV and/or AIDS are some of the challenges faced in practice (Blackwell & Littlejohns, 2010; Singh *et al*., 2015). Inexperienced clinicians may benefit from structured guidance provided by the NFAS in a resource-restrained context where patient prioritisation is key. The inherent limitations of the Delphi method include judgements of a select panel which may not be representative of the opinions of all clinicians. The time-consuming nature of participation which may impact on the thoroughness of the panel members’ responses, may also be a limitation. The final version of the NFAS reflects relevant areas of neonatal feeding prominently. The item selection clearly indicates the wide array of skills and components forming the foundation of neonatal feeding behaviour and responses that should be included in a comprehensive assessment instrument to be used by SLTs. The final content and checklist format of the NFAS was compiled as the first step in validating the NFAS and will be used in a future study to determine the preliminary psychometric properties of this instrument.
